# Randomized controlled trial of an alternative drainage strategy vs routine chest tube insertion for postoperative pain after thoracoscopic wedge resection

**DOI:** 10.1186/s12871-022-01569-w

**Published:** 2022-01-18

**Authors:** Shoucun Wei, Guangyan Zhang, Jue Ma, Lidan Nong, Jiatao Zhang, Wenzhao Zhong, Jianxiu Cui

**Affiliations:** 1 Department of Anaesthesiology, The First Affiliated Hospital of Jinan University, Guangzhou, China 510632; 2 Department of Anaesthesiology, Guangdong Provincial People’s Hospital, Guangdong Academy of Medical Sciences, Guangzhou, China 510080; 3 Guangdong Lung Cancer Institute, Guangdong Provincial People’s Hospital, Guangdong Academy of Medical Sciences, Guangzhou, China 510080

**Keywords:** Thoracoscopic wedge resection, Chest tube, Double-lumen central venous catheter, Postoperative pain

## Abstract

**Background:**

Thoracoscopic surgery has greatly alleviated the postoperative pain of patients, but postsurgical acute and chronic pain still exists and needs to be addressed. Indwelling drainage tubes are one of the leading causes of postoperative pain after thoracic surgery. Therefore, the aim of this study was to explore the effects of alternative drainage on acute and chronic pain after video-assisted thoracoscopic surgery (VATS).

**Methods:**

Ninety-two patients undergoing lung wedge resection were selected and randomly assigned to the conventional chest tube (CT) group and the 7-Fr central venous catheter (VC) group. Next, the numeric rating scale (NRS) and pain DETECT questionnaire were applied to evaluate the level and characteristics of postoperative pain.

**Results:**

NRS scores of the VC group during hospitalization were significantly lower than those of the CT group 6 h after surgery, at postoperative day 1, at postoperative day 2, and at the moment of drainage tube removal. Moreover, the number of postoperative salvage analgesics (such as nonsteroidal anti-inflammatory drugs [(NSAIDs]) and postoperative hospitalization days were notably reduced in the VC group compared with the CT group. However, no significant difference was observed in terms of NRS pain scores between the two groups of patients during the follow-up for chronic pain at 3 months and 6 months.

**Conclusion:**

In conclusion, a drainage strategy using a 7-Fr central VC can effectively relieve perioperative pain in selected patients undergoing VATS wedge resection, and this may promote the rapid recovery of such patients after surgery.

**Trial registration:**

ClinicalTrials.gov, NCT03230019. Registered July 23, 2017.

## Background

Favourable pain management after thoracic surgery is of great significance to prevent chronic pain and complications [1–3]. The current incidence of postoperative pain remains high, with evidence suggesting a 59–90% incidence of postoperative pain [4–6], and 11–35% of patients undergoing video-assisted thoracoscopic surgery (VATS) develop chronic pain postoperatively [7–11]. Many factors contribute to the occurrence of postoperative pain; compared with thoracotomy, postoperative pain after VATS is reduced. In addition, intercostal nerve injury, indwelling thoracic drainage tubes and psychosocial factors are also causes of postoperative pain [12–14]. Among them, the importance of chest drains in postoperative pain management is often overlooked, with limited attention focused on this aspect.

In recent years, with the popularization of enhanced recovery after surgery (ERAS), the tubeless strategy [15, 16] has been increasingly favoured by surgeons and patients. Thus, seeking a safe alternative to conventional chest tubes (CTs) or even abolishing drainage tubes has become a mainstream trend for many surgeons in minimally invasive pulmonary surgery. In our previously reported study, we provided evidence demonstrating that the alternative of conventional chest drainage by a 7-Fr double-lumen central venous catheter [17] (CVC) along with a prophylactic air-extraction strategy [18] can be safely applied to patients under lung wedge resection through VATS without causing more postoperative complications.

Intriguingly, we speculated that the alternative conventional chest drainage strategy of 7-Fr double-lumen CVC may reduce postoperative pain after lung wedge resection through VATS in the present study. To verify this hypothesis, we designed a single-centre, prospective, and randomized controlled trial to explore the effects of different drainage methods on acute and chronic pain in patients after lung wedge resection under VATS.

## Materials and methods

### Ethics statement and registration

This prospective, single-centre, open-label, and randomized controlled trial was approved by the Ethics Committee of Guangdong Provincial People’s Hospital (No. GDREC 2017261H) and registered before patient enrolment at www.clinicaltrials.gov on July 23, 2017 (registration number: NCT03230019). All participants signed written informed consent forms before enrolment in the study.

### Inclusion and exclusion criteria

The subjects for the screening included patients who received lung wedge resection through VATS at Guangdong Provincial People’s Hospital, and their age was above 18 years. The preoperative exclusion criteria were as follows: (1) any unstable systemic diseases such as active infection, poorly controlled hypertension within 3 months, diabetes or unstable angina; (2) history of ipsilateral chest surgery; (3) preoperative chest X-ray showing pneumonia or atelectasis; (4) bleeding tendency; (5) administration of anticoagulant drugs; or (6) history of other chronic chest pain. Patients were excluded if they needed to receive segmentectomy or lobectomy, if they showed severe adhesions during the surgery, if they required further exploratory surgery, or if air leaks were detected during leak examinations (Fig. 2).

### Sample size

The present study aimed to validate that the drainage strategy of the VC group after lung wedge resection through VATS could ameliorate postoperative pain. A previous retrospective study [18] documented that the mean NRS scores of postoperative acute pain in the CT group and VC group were 3.4 and 2.3, respectively, while the standard deviations were 1.1 and 0.8, respectively. The bilateral α was 0.01, and the power was 95%, in which half of the participants were assigned to the CT group and the rest to the VC group. The loss of follow-up and refusal to follow-up was calculated as 20%. Moreover, thirty-eight patients each were included in the CT group and VC group, as quantified by PASS software (version 15.0; NCSS, Kaysville, UT, USA). Hence, the total number of participants was at least 76.

### Randomization and blinding

Patients were selected before operation. After surgical incision (before wedge resection), one surgical team member randomly generated codes by using simple random sampling and SAS statistical software (SAS Institute, Cary, NC, USA) to randomly assign patients to the VC group or conventional CT group in a 1:1 ratio. It was easy to identify the grouping of patients during follow-up, and the patients and investigators were not blinded to the group assignment.

### Anaesthesia and surgery

For all patients, general anesthesia was induced with midazolam, sufentanil, propofol and cisatracurium, then double lumen endobronchial tube intubation was performed, and maintained with sevoflurane inhalation, remifentanil infusion, and cisatracurium. Non-steroidal drug flurbiprofen axetil 50 mg and antiemetic drugs were given before skin incision. In the post-anaesthesia care unit (PACU), each patient received intravenous analgesia: sufentanil 150 μg, flurbiprofen 300 mg and antiemetic agents with a total of 75 ml and a background dose of 1 ml/h. Rescue analgesics were administered according to the patient’s pain level after returning to the ward. Salvage analgesic measures indicated that patients with mild pain were given nonsteroidal anti-inflammatory drugs (NSAIDs) (such as Celebrex 200 mg and flurbiprofen axetil injection 50 mg), patients with moderate pain were treated with tramadol (50–100 mg), and patients with severe pain were treated with morphine (7–10 mg).

**Fig. 1:**
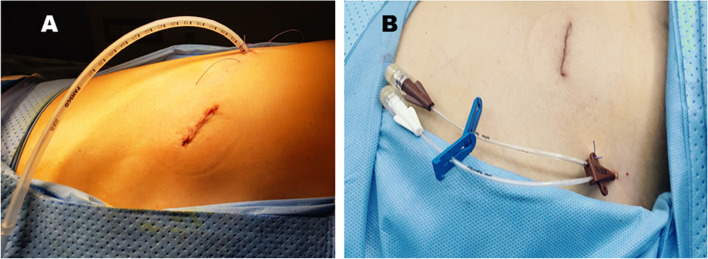
Procedure showing the position of the routine 20-Fr chest tube and 7-Fr double-lumen central venous catheter. **A** Insertion of the chest tube. **B** Insertion of the alternative thoracic drainage catheter

Before skin incision, 2% lidocaine was used for local infiltration anaesthesia along the incision. A three-centimetre-long incision was made between the anterior axillary line and the fourth or fifth intercostal line of the axillary line. Participants were excluded from the study in the case of severe adhesions or necessity of lobectomy due to insufficient surgical margins during the exploratory process. They were subjected to CT drainage, and all wedge resections were performed using a linear cutting stapler (Ethicon, Cincinnati, USA or Medtronic, Minneapolis, USA). After completion of wedge resection, a 20-Fr chest tube was inserted for drainage in the CT group (Fig. 1A), conducting an air-leak test and a water-sealed chest tube bottle was connected. Patients in the VC group were inserted a two-lumen central venous catheter (20 cm × 7 Fr) in the second intercostal space with a puncture needle. While the anaesthesiologist inflated the residual lung, the surgeon sutured the incision and conducted an air tightness test, and the CVC was clamped after air-extraction via injector (Fig. 1B). Patients with air leakage were excluded from the study and received CT drainage. All patients underwent a chest X-ray examination on the first day after surgery. Upon the observation of a large amount of pneumothorax, the CT group were required to strengthen deep breathing and perform coughing exercises or to undergo 8–10-cm H_2_O suction via the CT. In the scenario of massive pneumothoraxes in the VC group, a syringe was applied to perform a prophylactic air-extraction approximately 3 times a day through the CVC or the reinserted CT. CT/CVC extraction was considered upon indications of blood oxygen saturation (≥) 95%, fully dilated lungs, and no air leakage in the CT group.

### Pain evaluation

Chronic pain was defined as postoperative pain lasting longer than 3 months according to the International Association for the Study of Pain [11], and a numeric rating scale (NRS) was used to assess the postoperative pain level of the patients. A score of 0 indicated no pain, while a score of 10 represented the worst pain. On the basis of the evaluation results, the pain level was divided into 4 grades: no pain (NRS = 0), mild pain (NRS 1–3), moderate pain (NRS 4–6), and severe pain (NRS 7–10). The patient’s pain assessment was conducted in two stages, including the perioperative period and the assessment of chronic pain at 3 and 6 months after surgery. The perioperative evaluation referred to the evaluation during hospitalization and 1 month after the operation. A total of 7 pain evaluations were performed during hospitalization, which was the first 6 h after patients returned to the ward, 8 am and 6 pm on the first day after the surgery, 8 am and 6 pm on the second day after surgery, at the time of extubation and after the doctor issued a discharge from the hospital. In the follow-up of chronic pain, the pain DETECT questionnaire (PD-Q) was used to investigate the characteristics of the pain [19], which included allodynia, insufficiency, hyperalgesia, numbness, tingling, burning pain, and soreness.

### Study endpoints

The major endpoint of this study was the level of acute pain after surgery on the first day. The secondary observation indicators mainly included the duration of postoperative intravenous analgesia use, the frequency of postoperative salvage analgesics, the NRS score of extubation, the duration of postoperative drainage, postoperative hospitalization days, the NRS score of postoperative 1 month, and the level and characteristics of chronic pain of patients 3 and 6 months after surgery.

### Statistical analysis

Quantitative data are presented as the mean ± standard deviation (mean ± SD). Normally distributed data were compared between two groups using the unpaired *t*-test, and data with a skewed distribution were compared using the Mann-Whitney U test. Count data are presented as the actual number of cases and percentages, which were processed by the chi-square and Fisher’s exact tests. All the data were analysed by SPSS 25 software (IBM, Armonk, NY, USA), with *P* < 0.05 indicating statistical significance.

## Results

### Patient characteristics

**Fig. 2:**
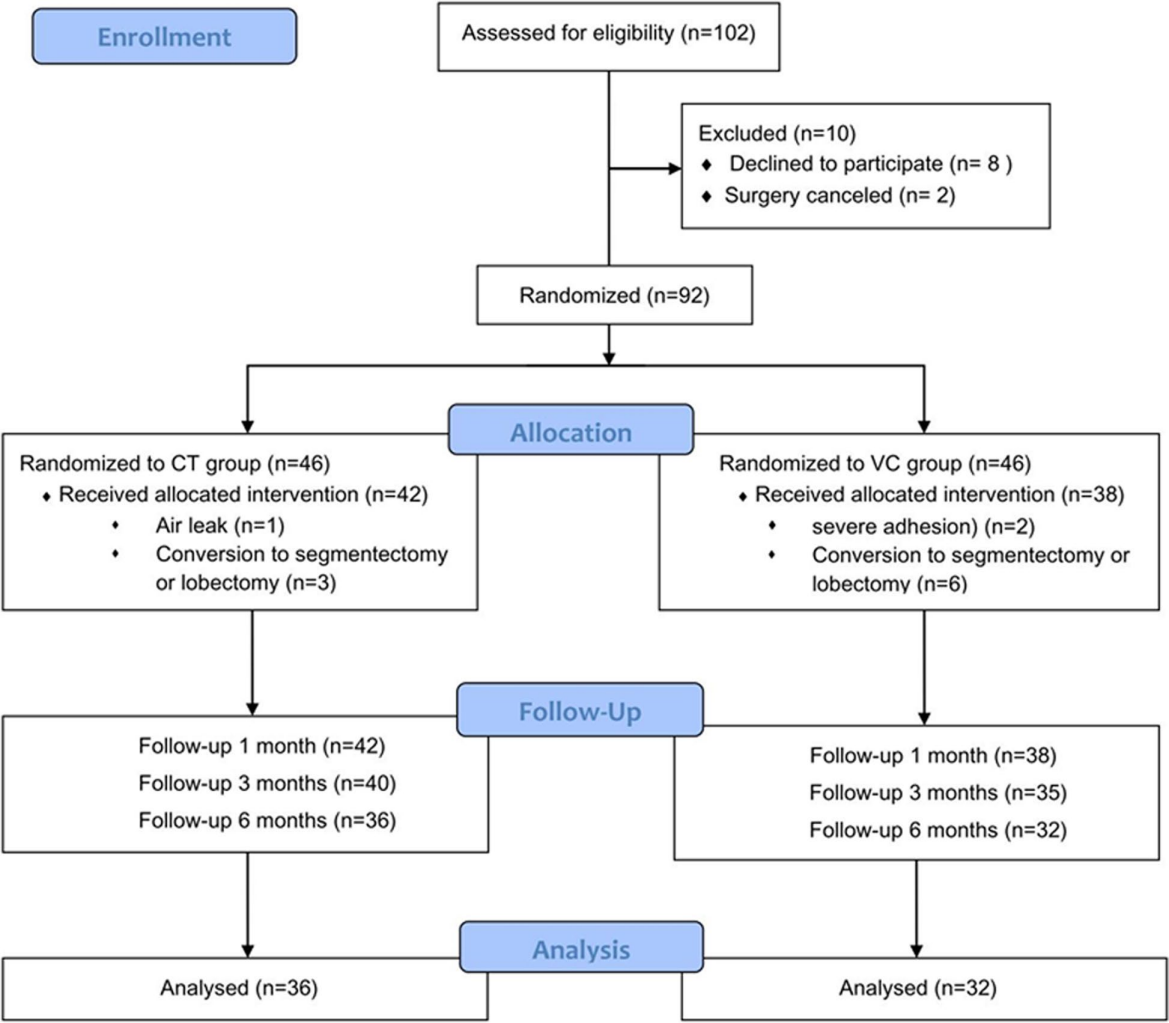
Consolidated Standards of Reporting Trials (CONSORT) flow diagram. CT, routine chest tube group; VC, central venous catheter group

From August 2017 to October 2018, a total of 102 patients with proposed wedge resection met the inclusion criteria for this study. Among them, 94 patients signed written informed consent forms, and the surgeries of 2 patients were temporarily cancelled; consequently, 92 patients were randomly assigned to the CT group and VC group. In addition, cases with intraoperative surgery type changes (*n* = 9), air leakage (*n* = 1), and severe adhesion (*n* = 2) were excluded. Finally, 42 patients were included in the CT group, and 38 patients were assigned to the VC group (Fig. 2). The demographic, ASA classification, NRS score baseline and operational details of both groups were balanced, and they are summarized in Table 1. No inpatient deaths or intensive care cases were identified.

**Table 1:** The demographic and clinical characteristics of the two groups

	CT group (*n* = 42)	VC group (*n* = 38)	*P* value
Age, years	54.7 ± 11.6	53.6 ± 9.2	0.646
Sex			0.644
Female	28	23	
Male	14	15	
BMI (kg/m^2^)	23.1 ± 2.4	22.3 ± 2.7	0.169
Classification of ASA			1.000
I	39	35	
II	3	3	
Surgical incision length, cm	3.6 ± 0.5	3.5 ± 0.6	0.713
Surgery time, median (25th–75th percentiles), minutes	60 (50–88)	60 (50–89)	0. 927
Resection length, median (25th–75th percentiles), mm	135 (120–180)	135 (120–180)	0. 636

Data are presented as the mean ± SD, median (range)

*CT* Chest Tube, *VC* Venous Catheter, *BMI* Body Mass Index, *ASA* American Society of Anesthesiologists

### Primary outcomes

The results of the primary outcome mainly focused on NRS scores of hospitalization. NRS scores of the VC group were considerably lower than those of the CT group at 6 h after surgery (2.3 ± 0.9 vs. 2.9 ± 0.8, *P =* 0.001), postoperative day 1 (2.6 ± 0.9 vs. 2.8 ± 0.8, *P =* 0.026), postoperative day 2 (2.2 ± 0.8 vs. 2.7 ± 0.7, *P =* 0.009), and CT removal (2.8 ± 0.7 vs. 3.3 ± 0.7, *P =* 0.003).

Moreover, the frequency of postoperative rescue analgesics NSAIDs (2.0 ± 1.5 vs. 3.0 ± 2.0, *P =* 0.023) and postoperative hospitalization days (2.7 ± 1.4 vs. 3.2 ± 1.2, *P =* 0.001) were remarkably reduced in the VC group compared with the CT group. No statistically significant difference was observed regarding the pain level of the two groups of patients at discharge (1.3 ± 0.7 vs. 1.5 ± 0.6, *P =* 0.267) (Table 2).

**Table 2:** Patients’ perioperative outcomes

	CT group (*n* = 42)	VC group (*n* = 38)	95% Confidence interval	*P* value
Postoperative 6 h (NRS)	2.9 ± 0.8	2.3 ± 0.9	–	0.001
POD1 (NRS)	2.8 ± 0.8	2.6 ± 0.9	–	0.026
POD1 NRS ≥3	19	11		
POD2 (NRS)	2.7 ± 0.7	2.2 ± 0.8	−0.75 to − 0.11	0.009
POD2 NRS ≥3	18	8		
Drainage tube removal (NRS)	3.3 ± 0.7	2.8 ± 0.7	–	0.003
Discharge (NRS)	1.5 ± 0.6	1.3 ± 0.7	−0.44 to 0.12	0.267
Intravenous analgesia days	1.3 ± 0.6	1.1 ± 0.4	−0.41 to 0.02	0.069
Sufentanil (μg)	54.6 ± 18.8	60.4 ± 26.6	−19.61 to 0.74	0.069
Flurbiprofen (mg)	109.0 ± 37.5	128.0 ± 53.2	−39.2 to 1.49	0.069
Salvage analgesics*				
NSAIDs	3.0 ± 2.0	2.0 ± 1.5	−1.72 to −0.13	0.023
Opioid	0.5 (0–1)	1.0 (1–2)	–	0.545
chest tube/catheter removal (days)	1.7 ± 0.7	1.7 ± 0.9	–	0.416
Length of stay (days)	3.2 ± 1.2	2.7 ± 1.4	–	0.001
POM1 (NRS)	1.1 ± 0.5	0.9 ± 0.5	–	0.182

*Frequency of salvage analgesics

Data are presented as the mean ± SD, median (range)

*POD* Postoperative Day, *NRS* Numeric Rating Scale, *NSAIDs* Nonsteroidal Antiinflammatory Drugs, *POM* Postoperative Month

It seems that the postoperative average NRS score was less than 3 in both groups. In fact, the proportion of patients with NRS scores≥3 on the first day and the second day (Table 2) in the CT group was higher than that in the VC group (44% vs 25%), so the demand for salvage analgesia NSAIDs in the CT group was greater than that in the VC group. However, there was no significant difference in terms of pain levels and characteristics between the two groups, including the frequency of postoperative salvage opioid use (*P =* 0.545), days of postoperative venous analgesia (1.1 ± 0.4 vs. 1.3 ± 0.6, *P =* 0.069).

### Secondary outcomes

Postoperative complications included pneumothorax, pleural effusion, chest tube reinsertion and subcutaneous emphysema. There were no differences between the two groups in these outcomes. Pneumothorax occurred in 4 patients (10.5%) in the VC group, and all patients recovered well after air extraction and did not need reinsertion of the CT. One patient (2.5%) in the CT group required CT reinsertion because of pleural effusion, and this patient was discharged on postoperative day 13.

NRS scores of postoperative at 1 month was (0.9 ± 0.5 vs. 1.1 ± 0.5, *P =* 0.182) in VC group and CT group (Tables 2-3). In the chronic pain follow-up, both groups of patients had different degrees of abnormal feelings, which were manifested as sensory changes related to weather change, mainly including paraesthesia, numbness and tingling (Table 3). The degree of discomfort did not affect sleep and daily activities, and no patient needed long-term pain medication.

**Table 3:** Three- and 6-month follow-up for chronic pain assessments

	POM3			POM6		
	CT group (*n* = 40)	VC group (*n* = 35)	*P* value	CT group (*n* = 36)	VC group (*n* = 32)	*P* value
Chronic pain positive (%)	10 (25)	7 (20)	0.783	7 (19.4)	4 (12.5)	0.521
Pain characteristic						
Tingling (%)	2 (5)	3 (8.6)	0.659	2 (5.6)	1 (3.1)	1.000
Numbness (%)	3 (7.5)	2 (5.7)	1.000	3 (8.3)	1 (3.1)	0.616
Paraesthesia (%)	5 (12.5)	2 (5.7)	0.438	2 (5.6)	2 (6.3)	1.000

Data are presented as n (%)

*POM* Postoperative Month

## Discussion

Thoracotomy has been reported to cause trauma and severe pain, while surgical trauma has been considerably reduced due to the development of VATS [20]. Moreover, with the popularization of ERAS, more precise management is required for the perioperative period. In addition to minimally invasive surgery and multi-modal analgesia, optimizing the management of various tubes, or even abolishing urethral catheters, postoperative drainage tubes in certain types of surgery could be of great value in promoting rapid recovery after surgery.

The population baseline of the two groups were balanced and same was for surgical procedure, while the postoperative NRS score and hospital stay were decreased in the VC group. This is mainly due to the fact that patients in the alternative drainage group received a 7-Fr central VC, which is small in diameter, greatly reduces the discomfort associated with chest tube. Patients in the VC group undergoing lung wedge resection could even get out of bed on the day of the operation. On the other hand, the VC group patients did not need to carry a chest drainage bottle after surgery, promoting early postoperative activity, which in turn was beneficial for wound healing [21]. In terms of length of stay, chest intubation and pain caused by the CT may have been one of the factors resulting in a prolonged hospital stay in the CT group, together with pneumothorax, pleural effusion, subcutaneous emphysema and other complications. In addition, one patient in the CT group was hospitalized for more than 13 days, and another six patients in the CT group experienced poor wound healing, which may be part of the reason for the significant difference in hospital stay.

With regard to chronic pain, our research results suggested only that there was no difference in the effects of the two kinds of tubes on abnormal skin sensation within half a year after the operation. It is hard to conclude that thoracic drainage tubes have nothing to do with postoperative chronic pain. We need more conclusive evidence to explain the connection between CTs and postoperative chronic pain.

We acknowledge that the present study has limitations and that its restrictive inclusion criteria may impede the universality and applicability of the results. First, the diameter of the catheter is small, the drainage capacity for pleural effusion is limited, and the tube is easily blocked. To prevent pneumothorax and massive pleural effusion after surgery, postoperative X-ray examination and dynamic observation of the patients’ reactions were very important, especially at the beginning of the study. Second, our investigation focused on the data and observations at a single research centre, and the sample size was relatively limited. Last, to directly explain the contribution of CTs to postoperative acute and chronic pain, it is necessary to further explore the difference between patients without CTs and the control group of patients in a follow-up study.

## Conclusion

In conclusion, a drainage strategy using a 7-Fr central VC can effectively relieve perioperative pain in selected patients undergoing VATS wedge resection, and this may be beneficial to the rapid recovery of such patients after surgery.

## Data Availability

The datasets used and/or analysed during the current study are available from the corresponding author on reasonable request.

## Abbreviations

VATS: Video-Assisted Thoracic Surgery
CVC: central venous catheter
NRS: Numeric Rating Scale
BMI: Body Mass Index
ASA: American Society of Anaesthesiologists
PACU: Post-anaesthesia Care Unit
NSAIDs: Nonsteroidal Anti-inflammatory Drugs
ERAS: Enhanced Recovery after Surgery
BIS: Bispectral Index
